# Supplementary material for: Endothelial leakiness elicited by amyloid protein aggregation
Source: Nat Commun. 2024 Jan 19;15:613. doi: 10.1038/s41467-024-44814-1 (PMC10798980; doi:10.1038/s41467-024-44814-1)
Supplement: Supplementary file 1 — Supplementary Information [file 41467_2024_44814_MOESM1_ESM.pdf]

Supplementary Information

Endothelial Leakiness Elicited by Amyloid Protein Aggregation

Li and Ni et al.

List of Content

Supplementary Figures 1-26

Supplementary Tables 1-4

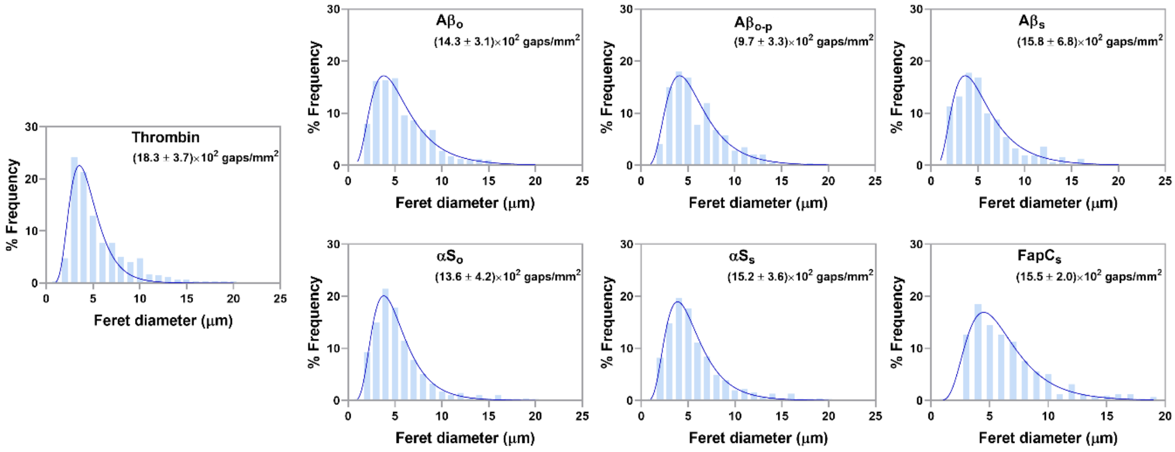

Supplementary Figure 1. Analyses of gap size and number after induced intercellular leakiness in HMVECs from treatment with various amyloid protein species. Confocal fluorescence images of induced gaps in HMVECs were analyzed with ImageJ software and the obtained data are expressed as frequency plots of the gaps' feret diameters. The label "o" stands for oligomers, "o-p" refers to early-stage protofibrils transitioning from the oligomers, and "s" for sonicated seeds.

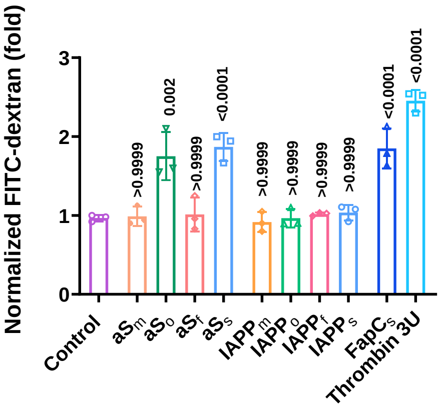

Supplementary Figure 2. Transwell assay quantitatively measured the endothelial leakiness after 30 min incubation with different peptide species. Thrombin (3 U/mL) acted as positive control of induced leakiness. Data are shown as mean ± SD (n = 3 biologically independent samples), analyzed via one-way ANOVA with Tukey's multiple comparison tests. The derived P values compared with control are shown.

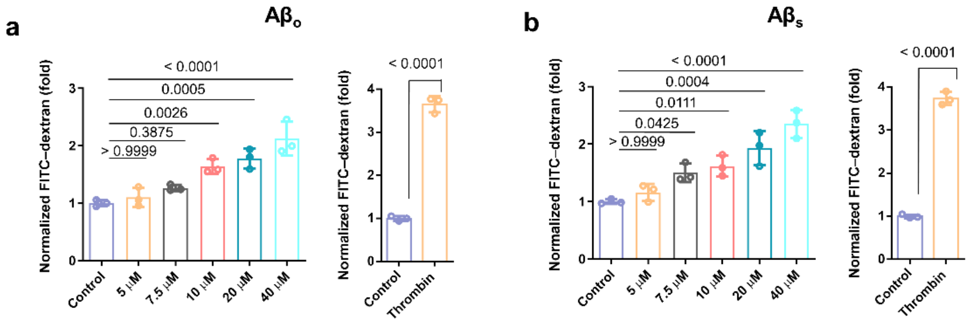

Supplementary Figure 3. Transwell assay quantitatively revealed the occurrence of endothelial leakiness in HMVECs treated with Aβ of different concentrations. After a 30 min incubation with two different forms of Aβ, **a** $A\beta_o$ and **b** $A\beta_s$, across a range of concentrations from 0 μM to 40 μM, leakiness across endothelial layer was quantified through transport of FITC-dextran, normalized against the control group. The degree of FITC-dextran transport was proportional to concentration of Aβ used. Thrombin (3 U/mL) acted as a positive control of induced leakiness. Data are shown as mean ± SD (n = 3 biologically independent samples), analyzed via one-way ANOVA with Tukey's multiple comparisons test, using GraphPad Prism. Derived P values when compared with control, were inserted in the figure.

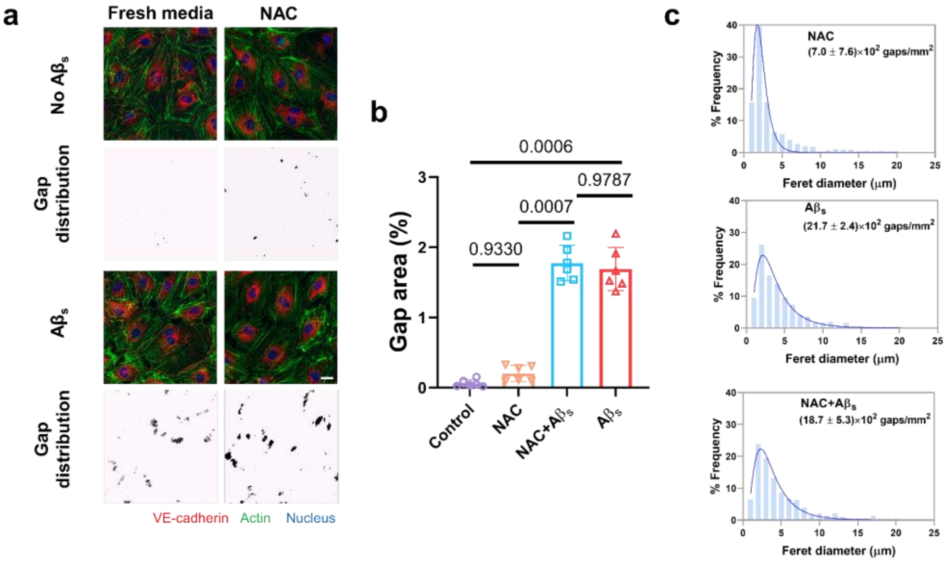

Supplementary Figure 4. Aβ-induced APEL is independent of increase in ROS production. **a** No significant reduction in leakiness (gap formation) was observed after $A\beta_s$ treatment, with or without NAC (10 mM, 1 h pre-treatment) (n = 3 biologically independent experiments). Red: VE-cadherin, green: actin, blue: nucleus. **b** Gap area percentages were analyzed via ImageJ according to the images related to panel **a**. Data are shown as mean ± SD (n = 6 biologically independent micrographs over 3 independent experiments), analyzed via one-way ANOVA with Tukey's multiple comparison tests. The derived P values compared with control were inserted in the panel. **c** Analysis of gap size and number after HMVECs were subjected to ROS scavenger treatment. With reference to panel **a**, NAC represents group treated with only N-acetylcysteine (NAC), $A\beta_s$ represents only $A\beta_s$, while NAC+$A\beta_s$ includes pre-treatment of NAC. Raw confocal images were analyzed via ImageJ software and gap distributions obtained are displayed as frequency plots of feret diameters.

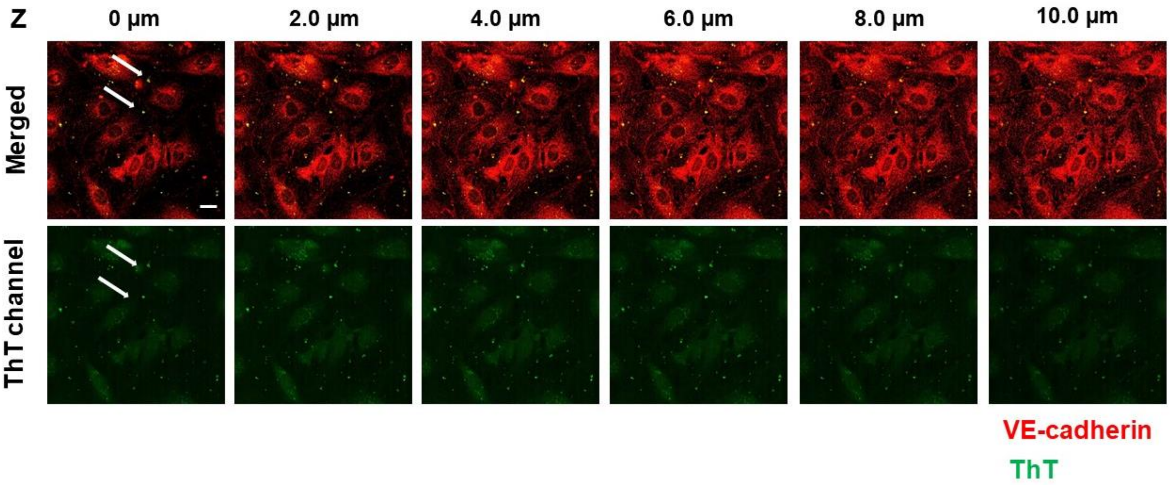

Supplementary Figure 5. Confocal Z-stacking further evidenced that Aβ_s were likely minimally internalized during the APEL duration of exposure. Z-stacked confocal images of HMVECs treated with ThT-labeled Aβ_s (20 µM, 30 min), in the same window of view, with Z-position downward from the higher plane (relatively defined as Z = 0 µm) to lower planes (n = 3 biologically independent experiments). Red: VE-cadherin, green: ThT-labeled Aβ_s. White arrows point to examples of ThT-labeled Aβ_s particles. Scale bar: 20 µm.

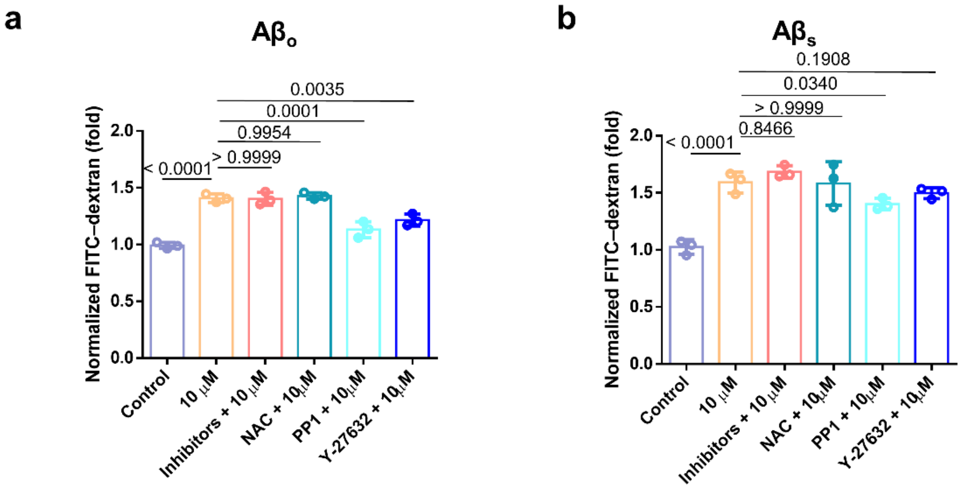

Supplementary Figure 6. The lower concentration of 10 µM of Aβ could still induce leakiness under key APEL-relevant assays. HMVECs were treated with either endocytosis inhibitors (5 mM MβCD and 10 µM MDC), ROS inhibitor (5 mM NAC), Src kinase inhibitor PP1 (10 µM), or ROCK inhibitor Y-27632 (10 µM) for 1 h prior to further treatment with 10 uM of **a** Aβ_o or **b** Aβ_s in a transwell assay. The treatments of MβCD, MDC or NAC did not significantly decrease the fold of FITC-dextran penetration compared to the respective counterparts without inhibitor treatments. However, PP1 and Y-27632 significantly reduced the fold of FITC-dextran penetration compared to their respective counterparts without inhibitor treatments. Data are expressed as means ± SD (n = 3 biologically independent samples), analyzed via one-way ANOVA with Tukey's multiple comparison tests. The derived P values compared between groups are presented in the panel.

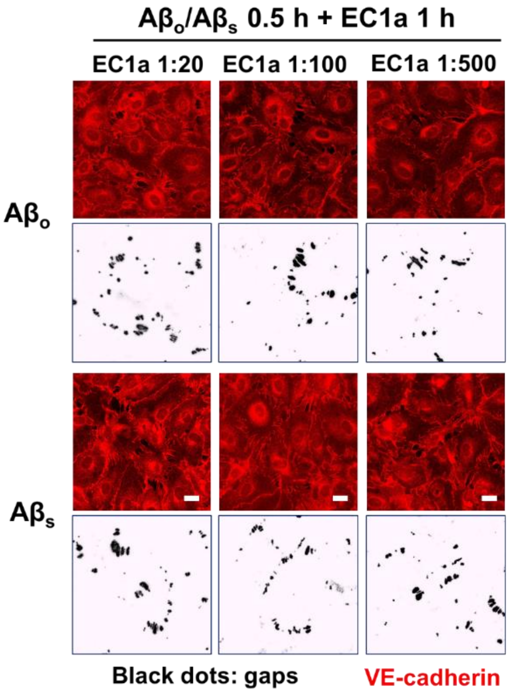

Supplementary Figure 7. Extracellular interactions of Aβ aggregates and VE-cadherin. Addition of $A\beta_o/A\beta_s$ for 0.5 h, followed by EC1 domain antibody (EC1a) treatment of different dilutions (1:20, 1:00 or 1:500) for 1 h (n = 3 biologically independent experiments). Black dots in the images represent holes in the HMVECs monolayer. Red: VE-cadherin. Scale bars: 20 µm.

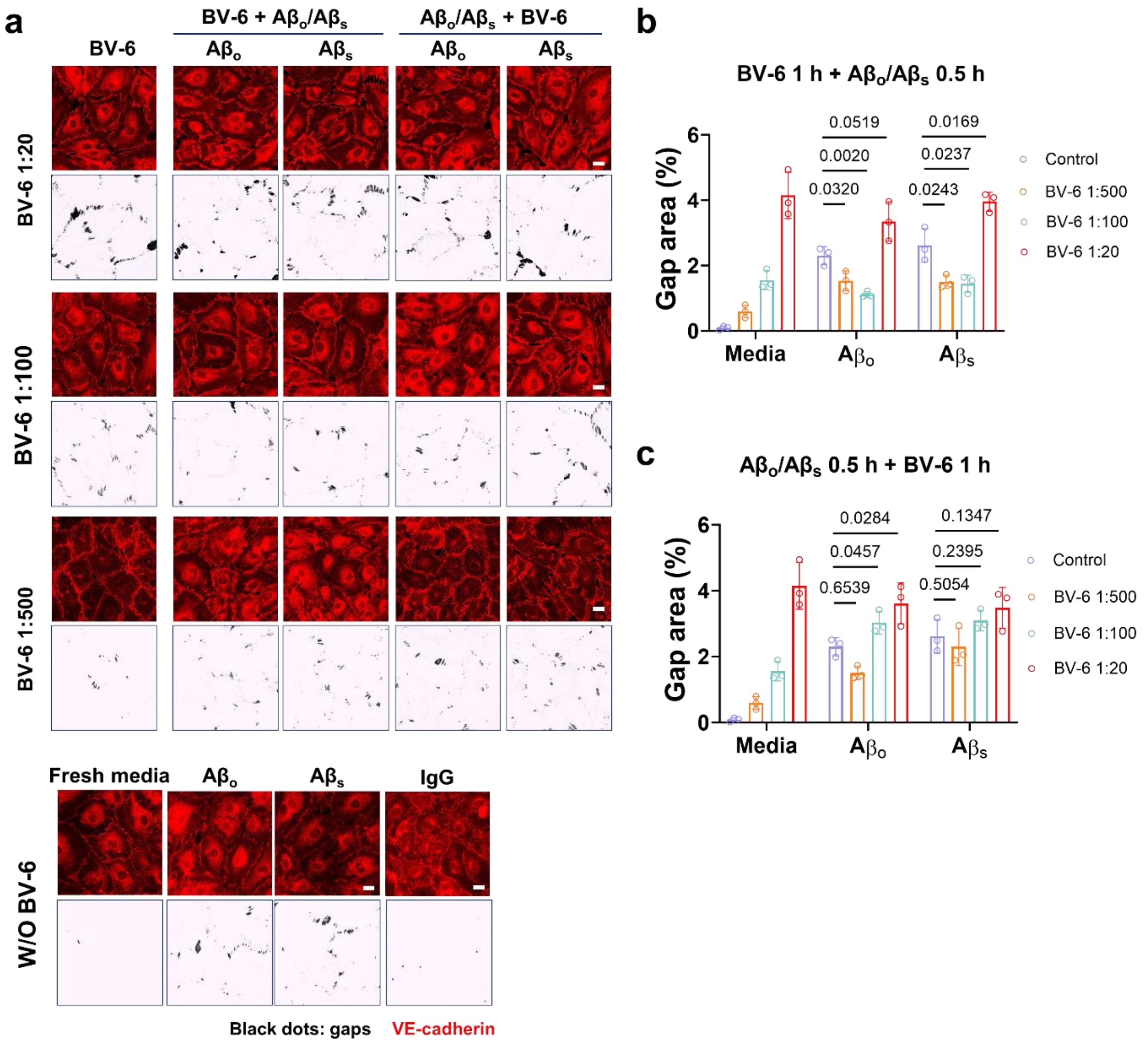

Supplementary Figure 8. Extracellular interactions of Aβ and VE-cadherin. **a** Different dilutions of BV-6 antibody (1:20, 1:100 or 1:500) pre-incubated with HMVECs 1 h prior to amyloid proteins treatment (BV-6+$A\beta_o/A\beta_s$). Addition of $A\beta_o/A\beta_s$ for 0.5 h, followed by BV-6 antibody treatment of different dilutions for 1 h ($A\beta_o/A\beta_s$+ BV-6) (n = 3 biologically independent experiments). There was no EL occurrence under the treatment of IgG with 1 µg/mL for 1 h. Gap distributions were obtained from captured confocal images through trainable Weka segmentation plugin in ImageJ software. Black dots in the images represent holes in the HMVECs monolayer. Red: VE-cadherin. Scale bars: 20 µm. **b, c** Gap area percentages were analyzed by ImageJ according to the images from the panel **a**. Data are shown as mean ± SD (n = 3 biologically independent micrographs over 3 independent experiments), analyzed via via two-tailed Student's t-tests. The derived P values compared between groups were inserted in the panels.

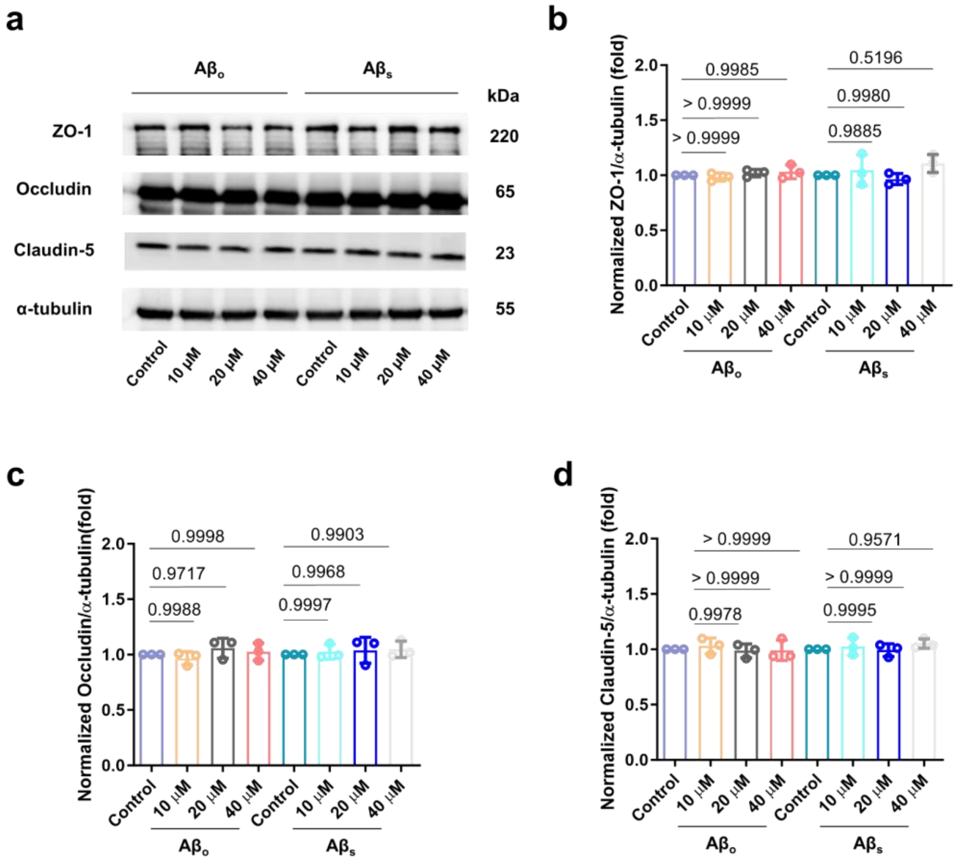

Supplementary Figure 9. Aβ-induced APEL was not observed to involve significant changes in the activities of tight junction signaling. a $A\beta_o$ and $A\beta_s$ treatments (0, 10 μM, 20 μM and 40 μM; 30 min) on HMVECs were subjected to immunoblotting analysis. The subsequent semi-quantitative analysis did not yield significant changes in expression of **b** ZO-1 (zonula occludens protein 1), **c** occludin and **d** claudin-5 proteins, which are key components of tight junctions. Data are presented as mean ± SD, n = 3 (biologically independent samples, with representative blots presented here), analyzed via one-way ANOVA with Tukey's multiple comparison tests.

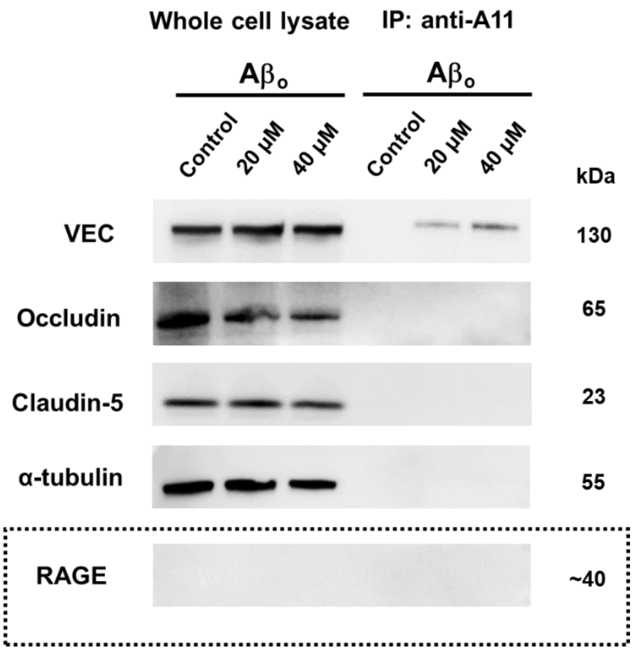

Supplementary Figure 10. Interactions of tight junction proteins with Aβ_o were not detected during the APEL process. Co-immunoprecipitation assay with anti-A11 antibody as an immunoprecipitant affirmed interactions between VE-cadherin (VEC) and Aβ_o following HMVECs' exposure to Aβ_o (0, 20 and 40 µM, 30 min). However, no detectable levels of interactions with tight junction proteins were found. Receptor for advanced glycation endproducts (RAGE) was additionally observed as not expressed in detectable levels for HMVECs. A representative blot is displayed, out of 3 biologically independent experiments.

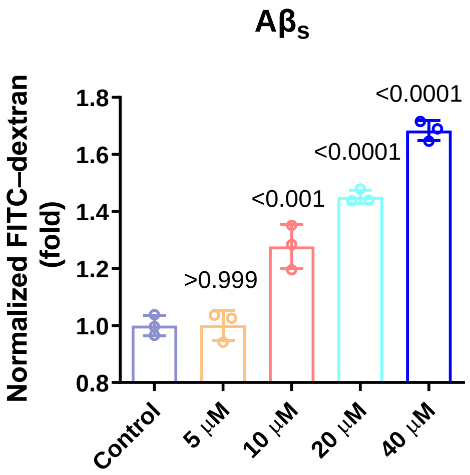

Supplementary Figure 11. Transwell assay on hCMEC/D3 brain endothelial cells treated with $A\beta_S$ revealed increased leakiness with increased concentrations. hCMEC/D3 were exposed to $A\beta_S$ from 0-40 μM, for the duration of 30 min. Penetration of FITC-dextran was used to measure permeability across cells. Results are presented as mean ± SD (n = 3 biologically independent samples), analyzed via one-way ANOVA with Tukey's multiple comparison tests. The derived P values compared with control are shown.

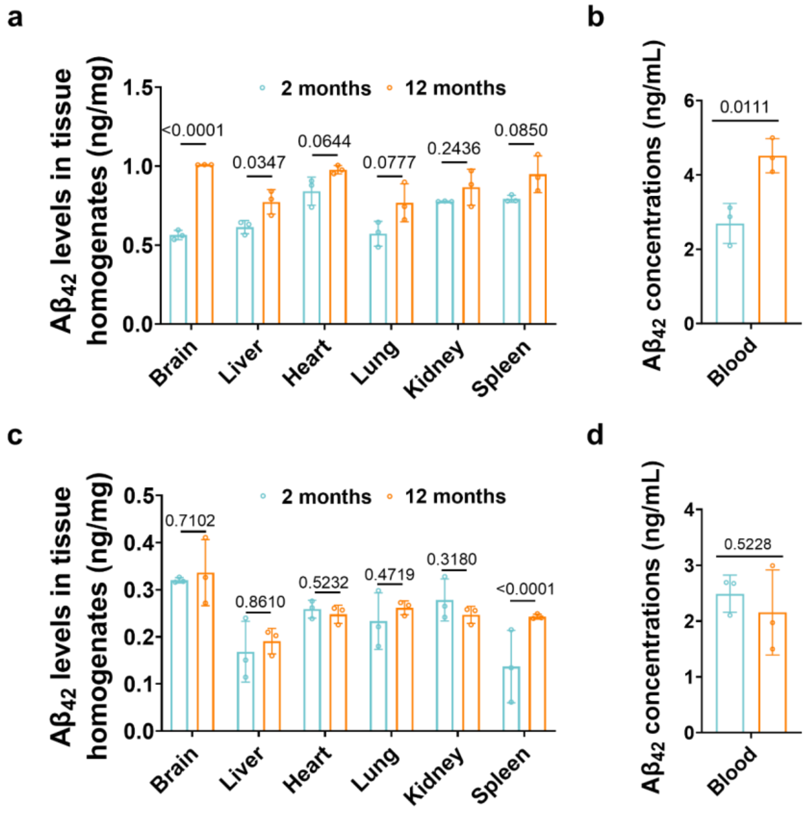

Supplementary Figure 12. Aβ$_{42}$ levels in the tissues and blood of APP/PS1 (a, b) and background strain mice C57BL/6J (c, d) mice at 2- and 12-months old, measured via a mouse Aβ$_{42}$ ELISA kit. The Aβ levels are normalized to the weight of the tissues or the volume of blood. Results presented are shown as mean ± SD (n = 3 animals), analyzed via two-tailed Student's t-tests. The derived P values compared between groups are shown.

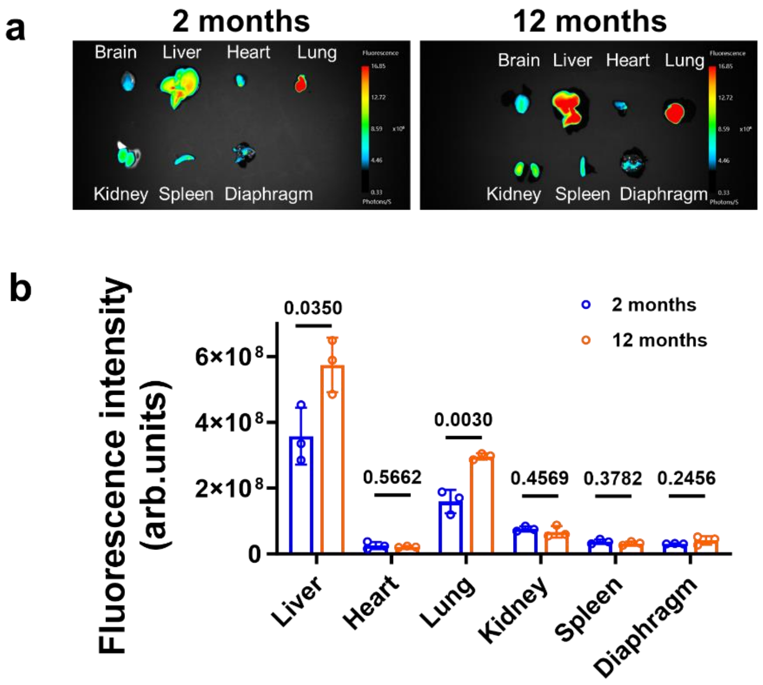

Supplementary Figure 13. Comparison of *in vivo* leakiness of APP/PS1 mice at 2- and 12-months old through measurement of EBD permeabilization 24 h post-injection. a Fluorescence imaging of the brain, the liver, the heart, the lungs, the kidneys, the spleen, and the diaphragm and **b** quantitative analysis of the tissues in APP/PS1 mice. Results presented are shown as mean ± SD (n = 3 animals), analyzed via two-tailed Student's t-tests. The derived P values compared between groups were presented in the panel.

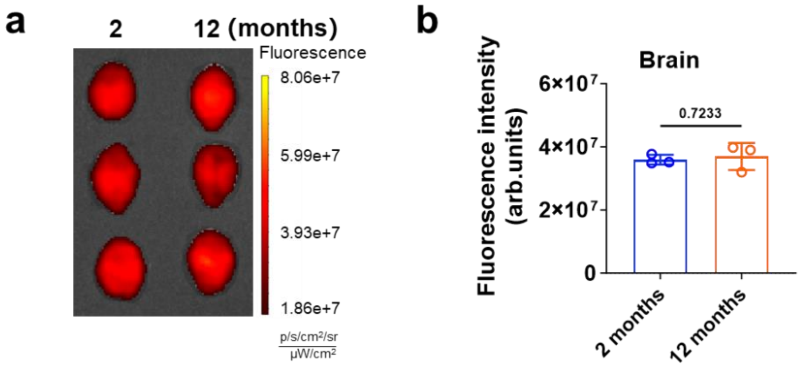

Supplementary Figure 14. Comparison of leakiness across the blood-brain barrier through a measurement of EBD permeabilization 24 h post-injection in background strain C57BL/6J mice of 2- and 12-months old. a Fluorescence imaging of the brain. **b** Quantification of fluorescence intensities for the brain. Results presented are shown as mean ± SD (n = 3 animals), analyzed via two-tailed Student’s t-tests. The derived P values compared between groups were presented in the panel.

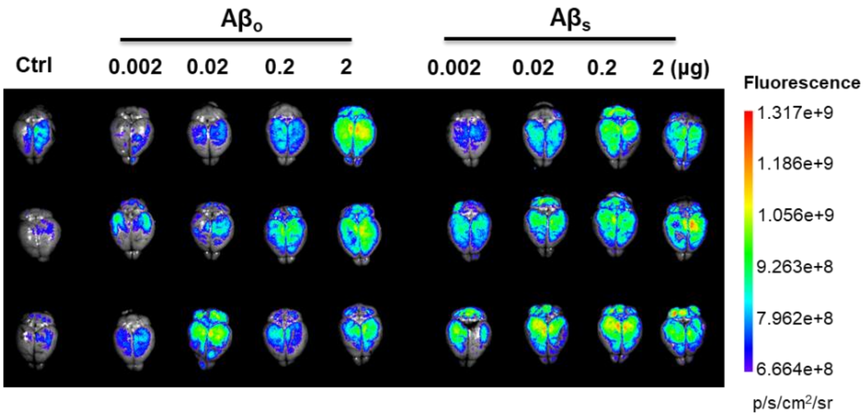

Supplementary Figure 15. ***In vivo*** **experiment involving injection of Aβ_s or Aβ_o (0.002, 0.02, 0.2 and 2 μg) in mice revealed increased leakiness across the blood-brain barrier through measurement of EBD permeabilization 24 h post-injection (n = 6 animals, including data for 3 female mice shown here and data for 3 male mice shown in Fig. 6c).**

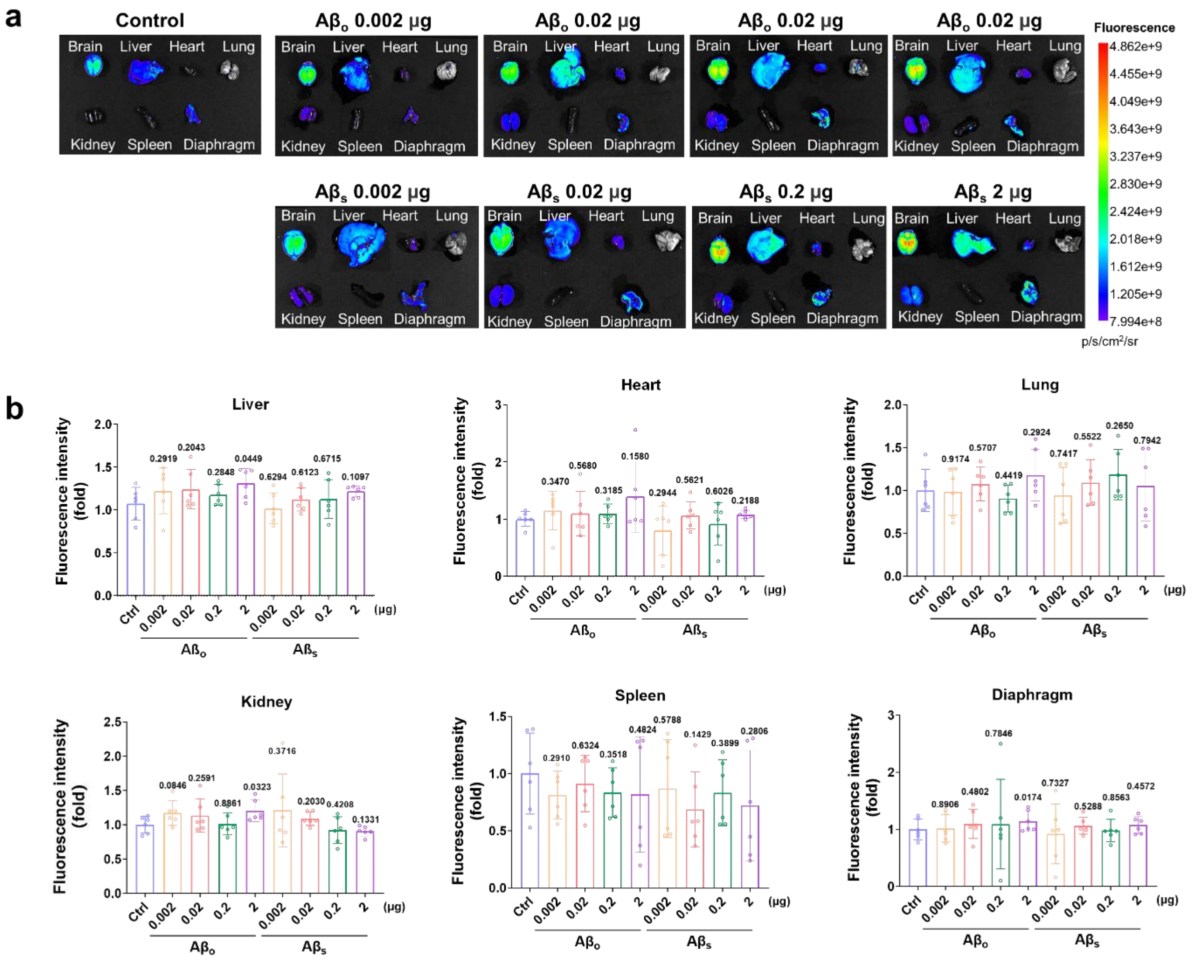

Supplementary Figure 16. ***In vivo*** **leakiness assay involving the injection of Aβ$_s$ or Aβ$_o$ (0.002, 0.02, 0.2 and 2 μg) into mice through measurement of EBD permeabilization 24 h post-injection. a** Fluorescence imaging of different tissues, including the brain, the liver, the heart, the lungs, the kidneys, the spleen, and the diaphragm. **b** Quantification of fluorescence intensities for tissues. Results presented are shown as mean ± SD (n = 6 animals), analyzed via two-tailed Student's t-tests. The derived P values compared with control were presented in the panel.

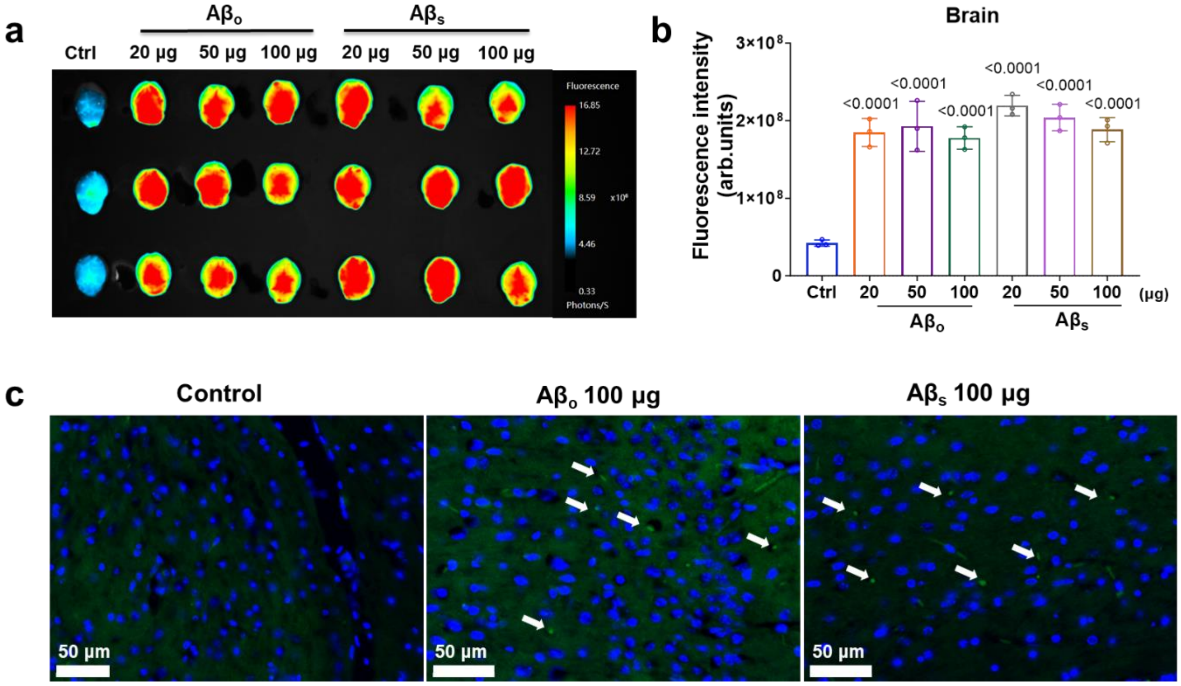

Supplementary Figure 17. Aβ-induced endothelial and vascular leakiness *in vivo*. a, b $A\beta_o$ or $A\beta_s$ with 20, 50 and 100 μg were injected into mice and the increased leakiness across the blood-brain barrier indicated by EBD's permeabilization was measured at 24 h post-injection. Results presented are shown as mean ± SD (n = 3 animals), analyzed via two-tailed Student's t-tests. P values compared with control are shown. **c** Immunofluorescence analysis of transverse brain sections of mice 24 h post-injection with $A\beta_s$ or $A\beta_o$ (100 μg) revealed presence of $A\beta_s$ or $A\beta_o$ (indicated with white arrows), compared to the absence of such structures in the brains of mice injected with only EBD. Anti-Aβ antibody (6E10) staining in green and DAPI in blue, with scale bars = 50 μm.

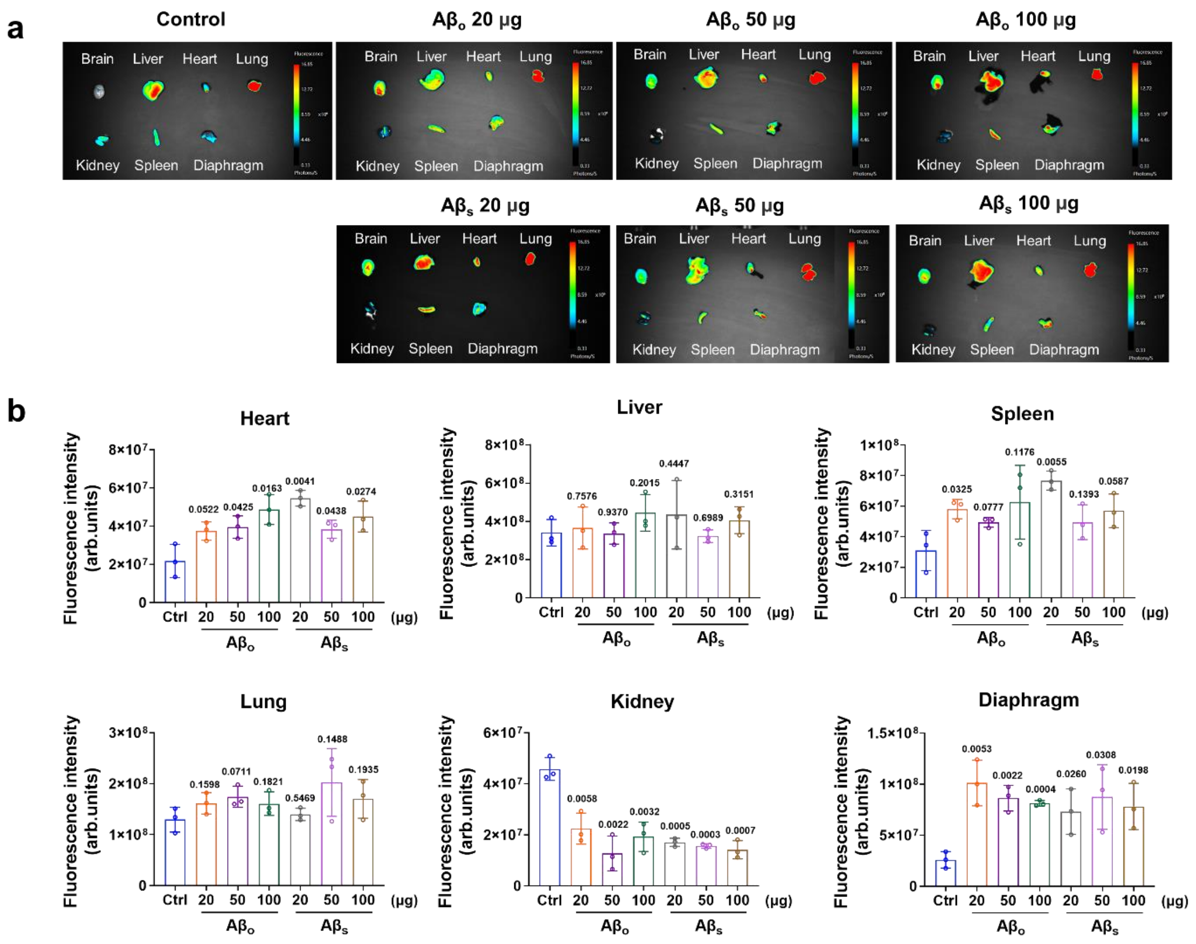

Supplementary Figure 18. ***In vivo*** **leakiness assay involving the injection of $A\beta_s$ or $A\beta_o$ (20, 50 and 100 μg) into mice through measurement of EBD permeabilization 24 h post-injection. a** Fluorescence imaging of different tissues, including the brain, the liver, the heart, the lungs, the kidneys, the spleen, and the diaphragm. **b** Quantification of fluorescence intensities for tissues. Results presented are shown as mean ± SD (n = 3 animals), analyzed via two-tailed Student's t-tests. The derived P values compared with control were presented in the panel.

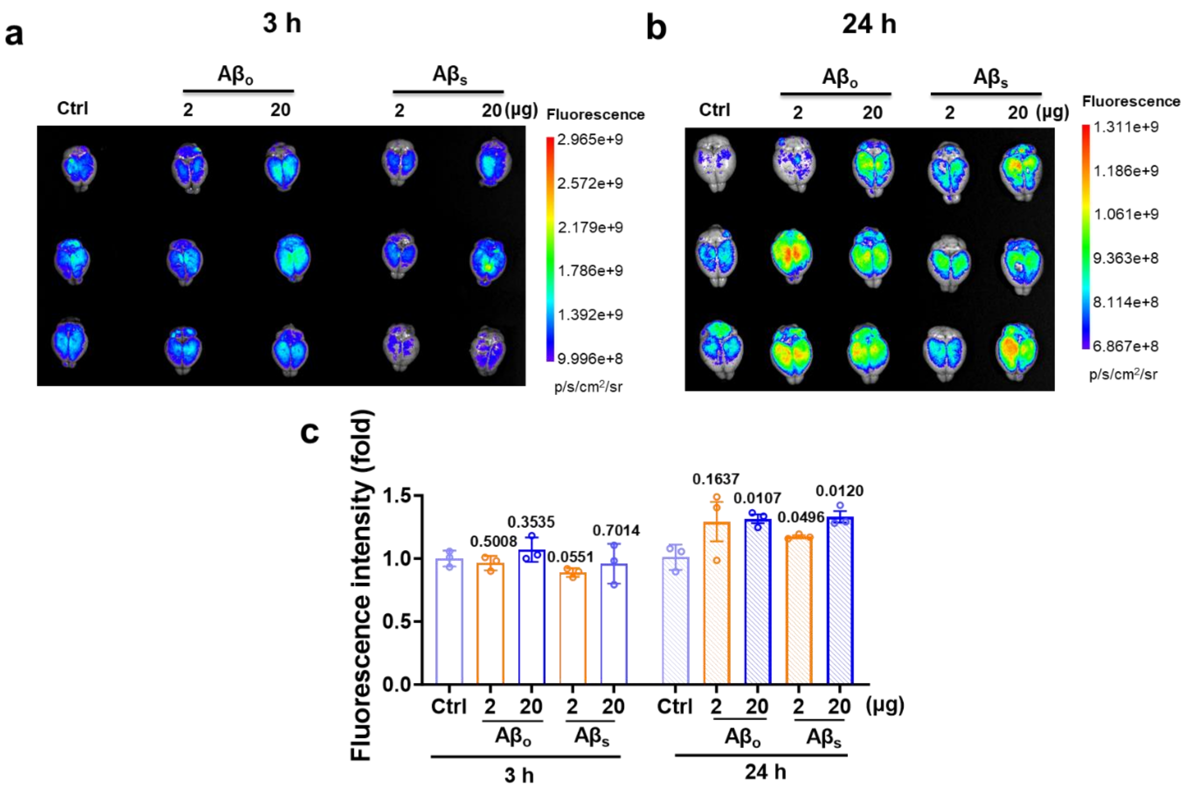

Supplementary Figure 19. Timeframe of Aβ-induced vascular leakiness *in vivo*. The FITC-dextran (10,000 Da) permeabilization after **a** 3 h or **b** 24 h injection of $A\beta_o$/$A\beta_s$ (2 and 20 μg) was used to indicate APEL occurrences in mice. FITC-dextran was injected 30 min before the measurement. **c** Quantification of fluorescence intensities for the brain organs from panels **a** and **b**. Results presented are shown as mean ± SD (n = 3 animals), analyzed via two-tailed Student's t-tests. The derived P values compared with control are presented in the panel.

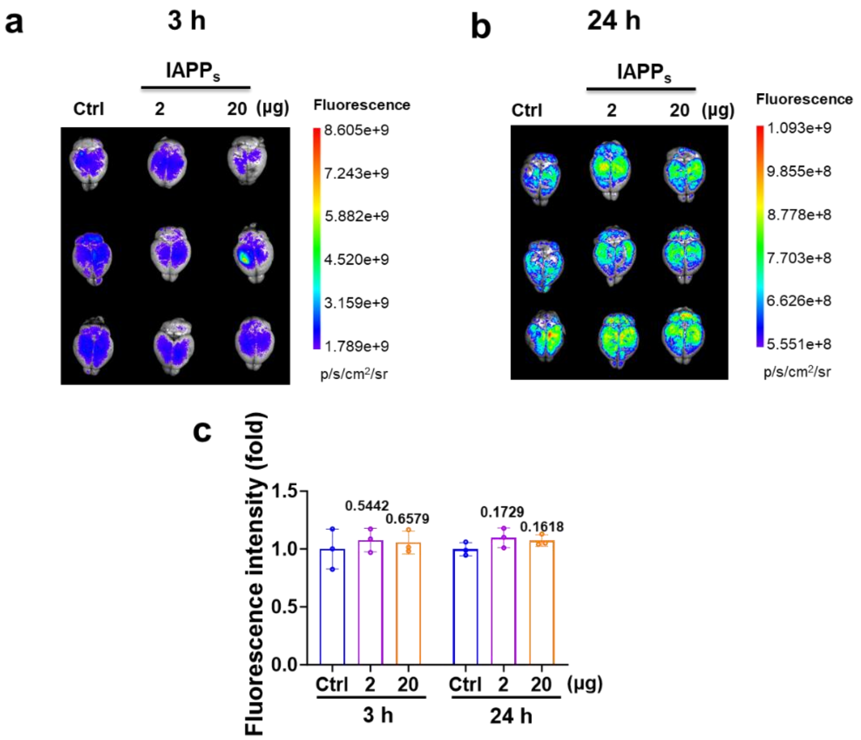

Supplementary Figure 20. Detection of IAPP-incited vascular leakiness *in vivo*. FITC-dextran (10,000 Da) permeabilization after **a** 3 h or **b** 24 h injection of $IAPP_S$ (2 and 20 μg) was used to indicate APEL occurrences in mice. FITC-dextran was injected 30 min before the measurement. **c** Quantification of fluorescence intensities for the brain organs from panels **a** and **b**. Results presented are shown as mean ± SD (n = 3 animals), analyzed via two-tailed Student's t-tests. The derived P values compared with control are presented in the panel.

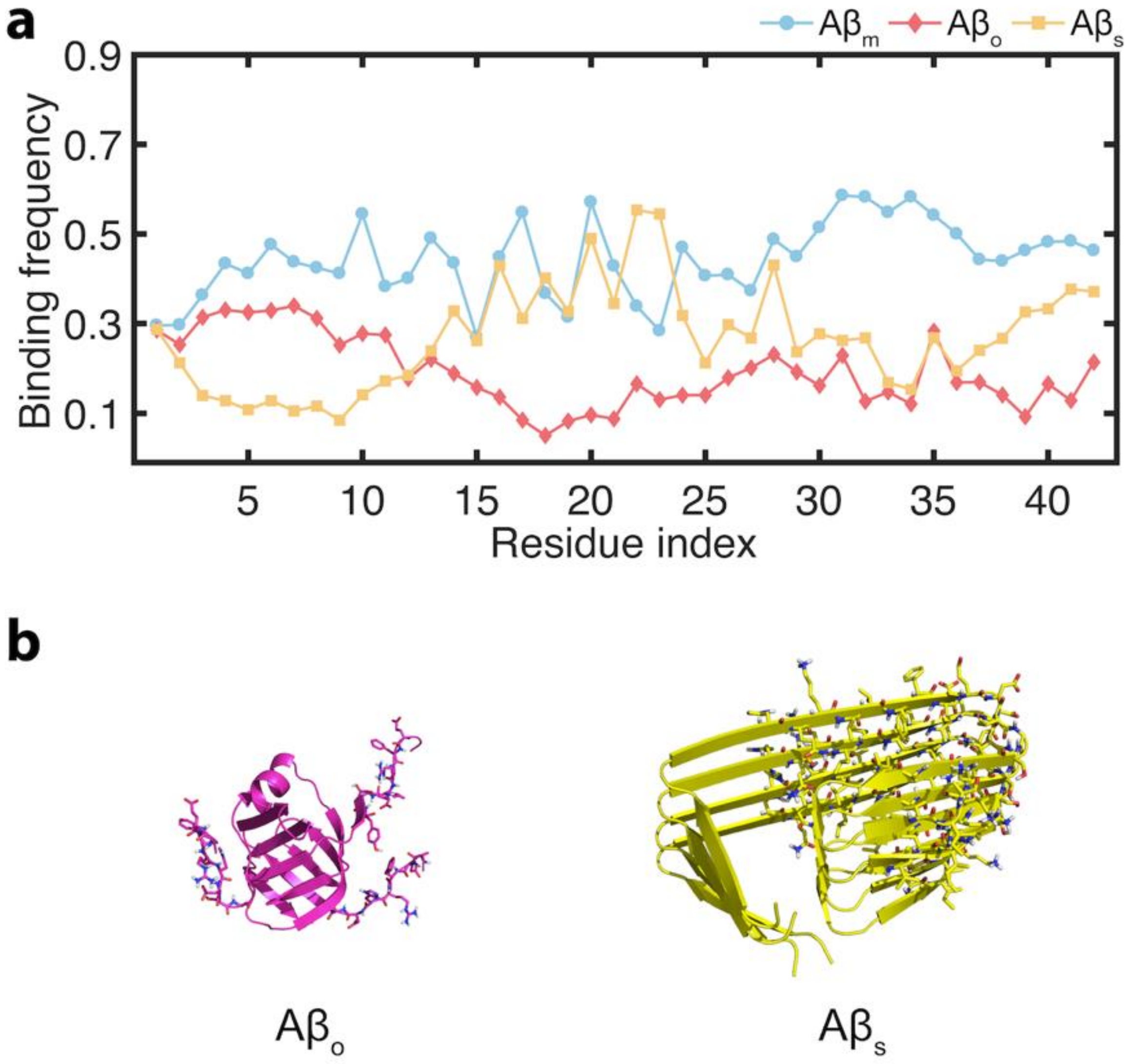

Supplementary Figure 21. Binding frequency of an EC1 dimer with an Aβ monomer (Aβ$_m$), an Aβ oligomer (Aβ$_o$) and an Aβ seed (Aβ$_s$), respectively. a The binding frequencies of the EC1 dimer with amyloid proteins were computed for the last 20 ns of the binding simulation. **b** Structures of Aβ$_o$ and Aβ$_s$ representing high binding frequencies over 0.3. The residues highly interacted with the EC1 dimer are represented as sticks. The β-sheets contents are notable in the structures of Aβ$_o$ and Aβ$_s$.

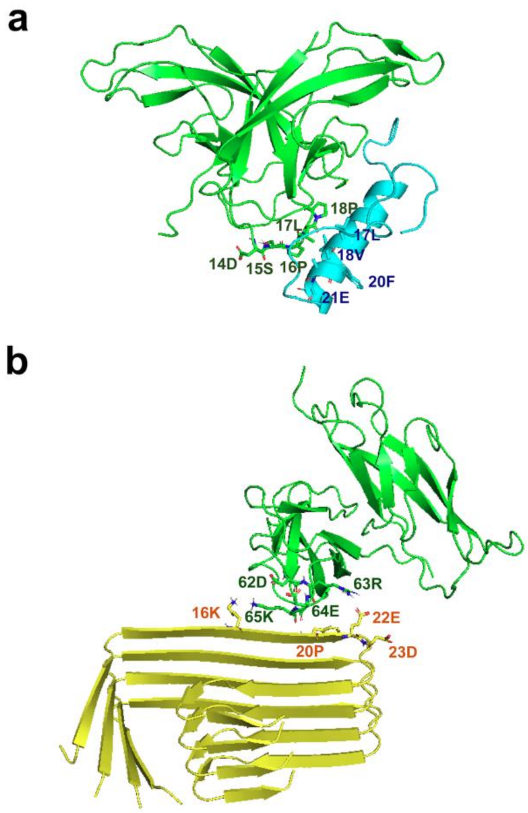

Supplementary Figure 22. Representative configurations of binding between Aβ species and EC1 dimer. The specific types of amino acid interactions between the Aβ species and EC1 dimer drove the binding. **A** Details of binding between EC1-dimer and $A\beta_m$. **b** Details between EC1 dimer and $A\beta_s$. Green, blue, and yellow sticks represent the residues in EC1 dimer, $A\beta_m$, and $A\beta_s$.

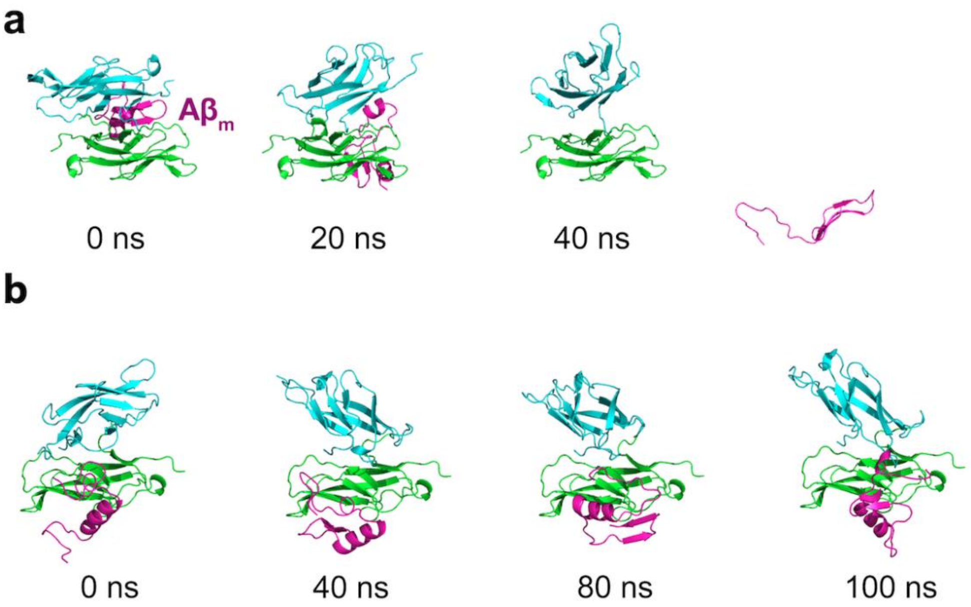

Supplementary Figure 23. Representative trajectories of an EC1 cadherin dimer with an Aβ monomer (Aβ$_m$). Two different trajectories of the EC1 dimer-Aβ$_m$ complex remained associated during the 100 ns of steered discrete molecular dynamics (sDMD) simulations. Structure details of the EC1 dimer and Aβ$_m$. Aβ$_m$ is represented in magenta color. Cyan and green colors indicate the flexible and immobilized domain of the EC1 cadherin, respectively. **a** The EC1 dimer was not dissociated because the Aβ$_m$ was unbound from the EC1 dimer after 40 ns of sDMD simulation. **b** The EC1 dimer was not dissociated even though the Aβ$_m$ was bound during the entire sDMD simulation.

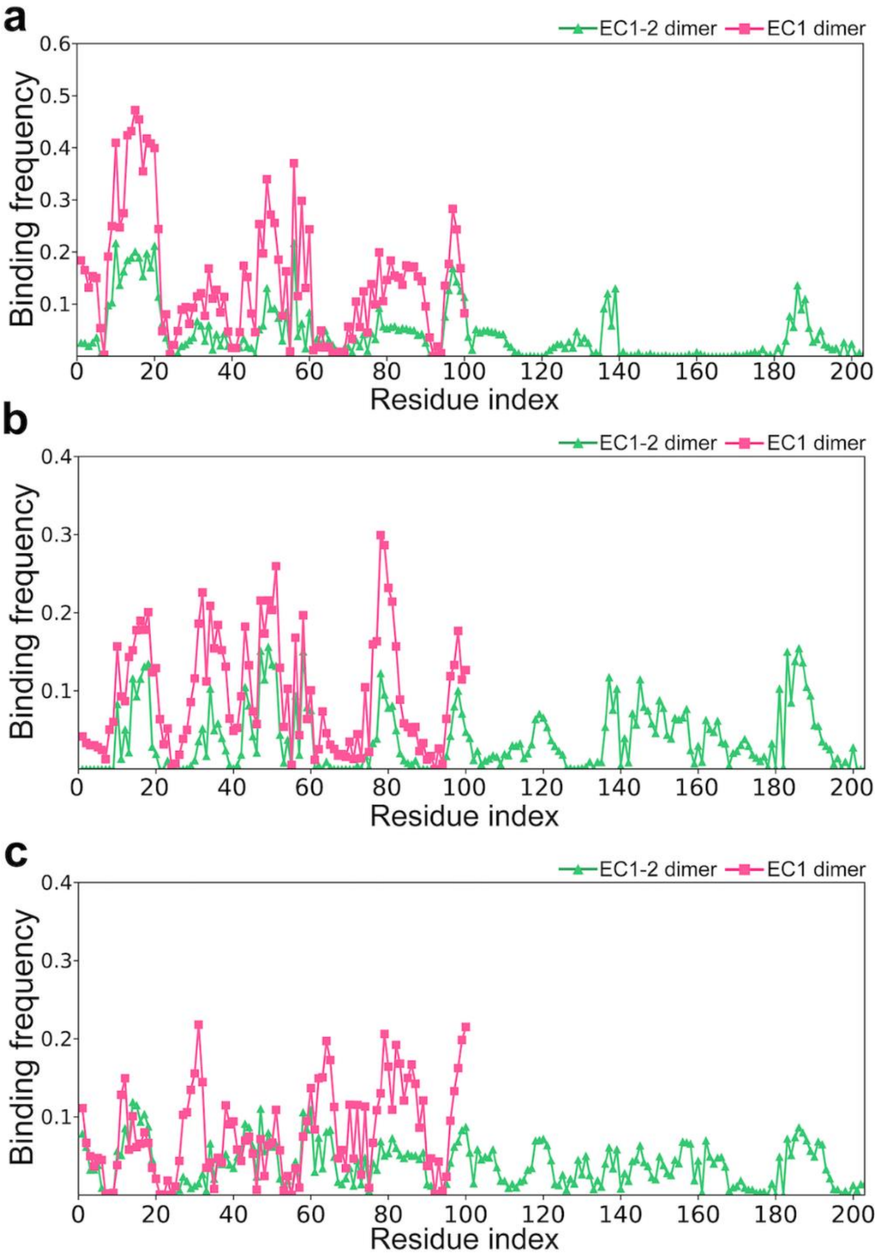

Supplementary Figure 24. Binding-frequency comparison of amyloid proteins with EC1 and EC1-2 cadherin dimers. Binding frequencies of the amyloids with EC1 dimer and EC1-2 dimer were marked as square and triangular, respectively. Binding frequencies with **a** a monomer ($A\beta_m$), **b** an oligomer ($A\beta_o$), and **c** a seed ($A\beta_s$). The results suggest that different Aβ species can bind nearly equally to different EC domains of VE-cadherin. The lower binding frequency for EC1-2 dimer resulted from approximately doubled binding sites compared to EC1 dimer.

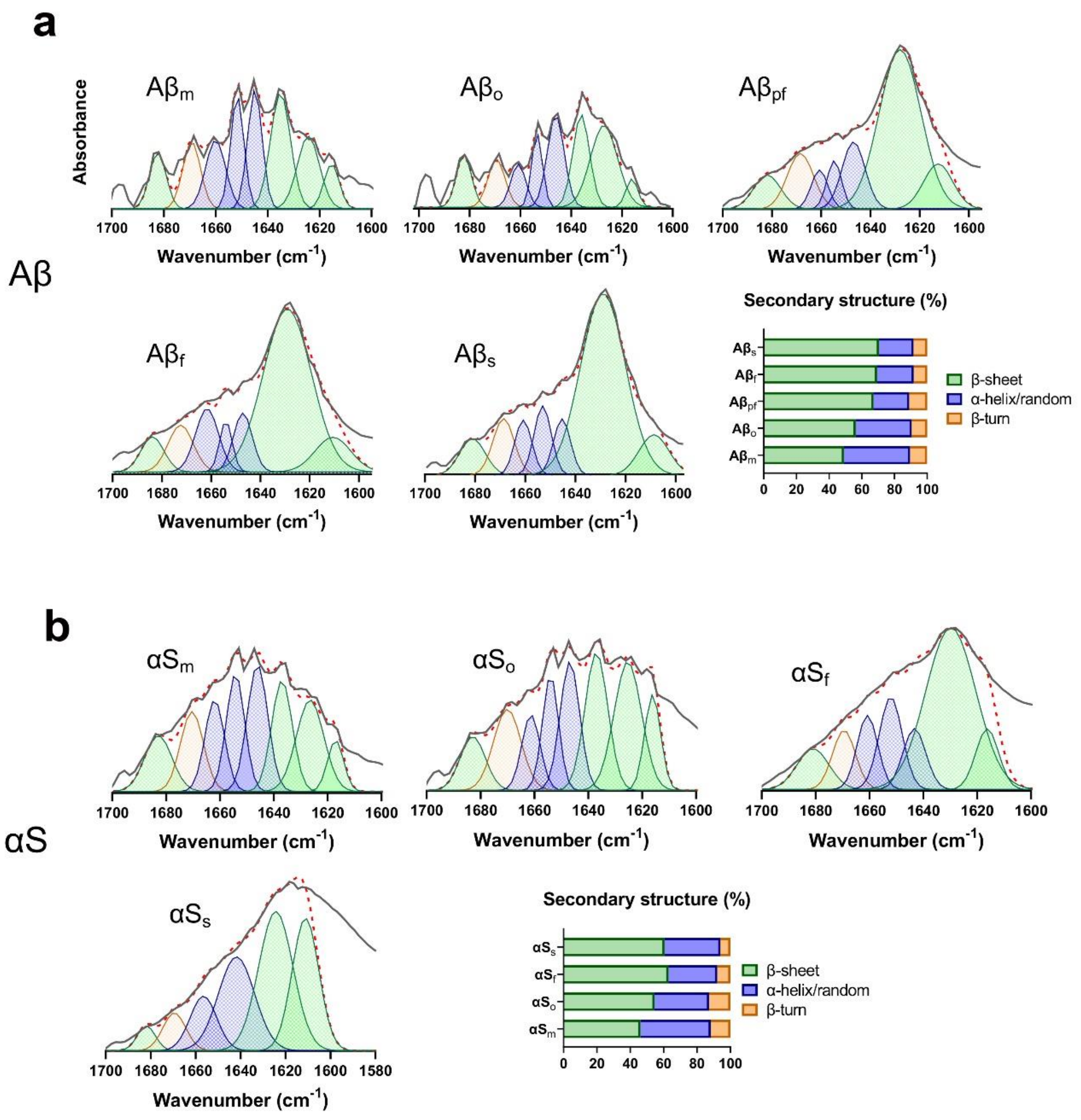

Supplementary Figure 25. Quantitative secondary analysis of Aβ and αS species obtained through ATR-FTIR spectroscopy based on the C=O stretching vibration of the peptide backbone in the amide I band region (1580-1700 cm^{-1}). a FTIR spectra of Aβ at different stages of aggregation (monomeric/oligomeric/protofibrillar/fibrillar) as well as in the form of amyloid seeds, showing a gradually increasing β-sheet content through fibrillization. The β-sheet content was comparable for $A\beta_f$ and $A\beta_s$. **b** FTIR spectra of monomeric/oligomeric/fibrillar αS and αS seeds. Each spectrum is accompanied with deconvoluted peaks revealing the secondary structure profile (%) (β-sheet, α-helix/disordered, β-turn) of each amyloid peptide at varying incubation timepoints, showing a gradually increasing β-sheet content through fibrillization. The β-sheet content was comparable for αS_f and αS_s. Secondary structure types and spectrum bands are highlighted accordingly (β-sheet as green, α-helix/disordered as blue, β-turn as orange, FTIR original spectrum as gray and deconvoluted area as dashed red). Deconvoluted band regions were assigned based on literature[1].

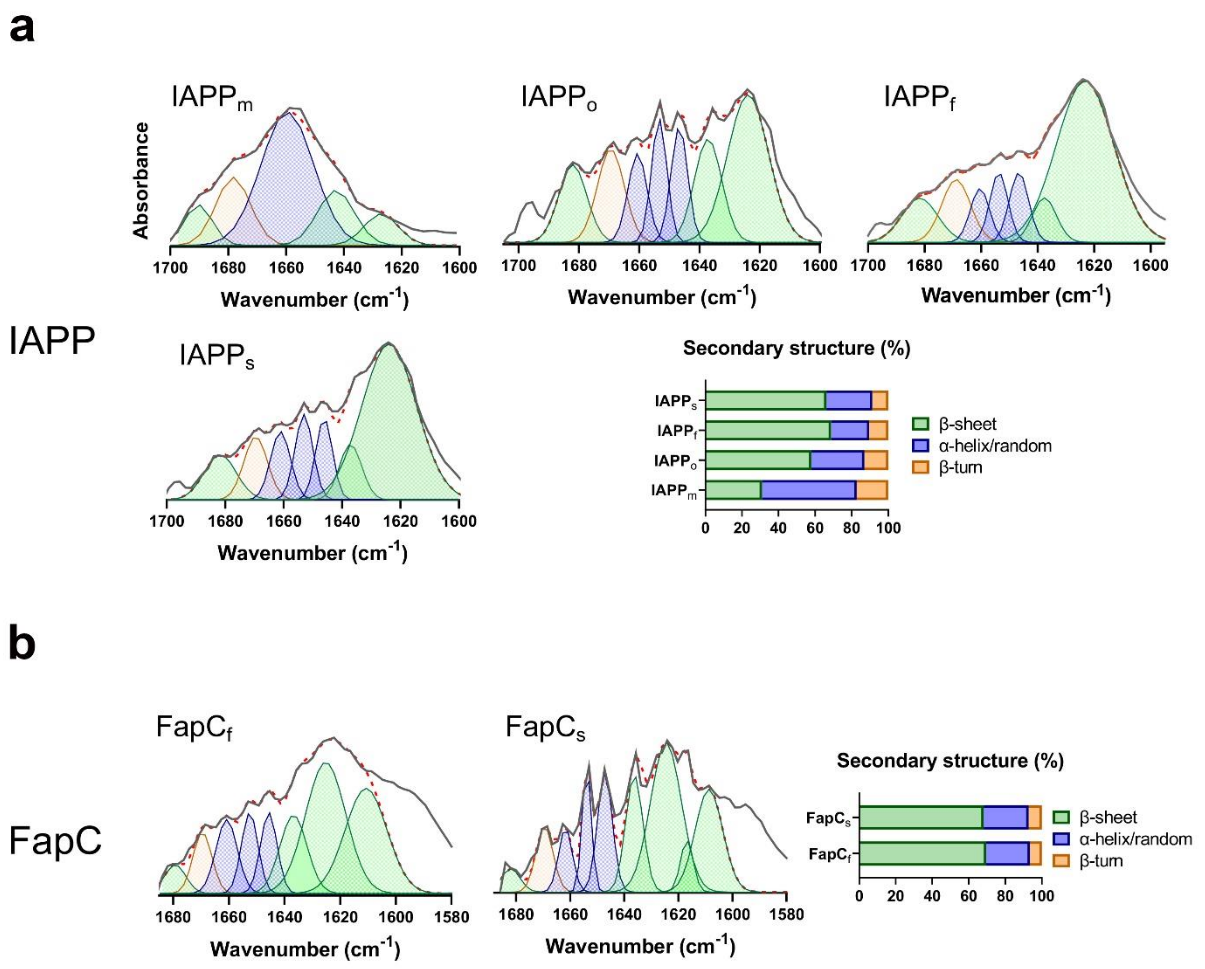

Supplementary Figure 26. Quantitative secondary analysis of IAPP and FαpC species obtained through ATR-FTIR spectroscopy based their equivalent C=O stretching vibration bands at the amide I band region (1580-1700 cm^{-1}). a FTIR spectra and secondary structure profile (%) of IAPP at different stages of aggregation (monomeric/oligomeric/fibrillar) as well as in the form of amyloid seeds, showing a gradually increasing β-sheet content through fibrillization. The β-sheet content was comparable for IAPP$_f$ and IAPP$_s$. **b** FTIR spectra and secondary structure profiles (%) of FapC fibrils and seeds. The β-sheet content was comparable for FapC$_f$ and FapC$_s$. FapC monomers have been reported by literature and our previous work to exhibit mainly a random coiled structure (~45%) combined with a significant α-helical content (~30%)[2, 3]. Coloring and deconvolution analysis were performed similarly as for Supplementary Figure 25.

Supplementary Table 1. Gap size analysis derived from confocal images of HMVECs treated with different amyloid proteins. The label "o" stands for oligomers, "o-p" refers to early-stage protofibrils transitioning from the oligomers, and "s" for sonicated seeds.

	**Median (μm)**	**Mean (μm)**	**Skewness**	**Coefficient of variation (cv)**
**Thrombin**	4.462	5.756	3.211	68.29%
**$A\beta_o$**	5.012	5.987	2.606	62.85%
**$A\beta_{o\text{-}p}$**	5.203	6.348	2.833	61.26%
**$A\beta_s$**	4.907	5.988	2.269	66.81%
**$FapC_s$**	5.817	6.844	2.440	58.88%

Supplementary Table 2. Antibodies used for immunoblotting experiments.

**Antibody**	**Product origin**
Anti-caspase 3	Cell Signaling Technology
Anti-caspase 9	Cell Signaling Technology
Anti-PARP	Cell Signaling Technology
Anti-phospho VE-cadherin (Y658)	Thermo Fisher Scientific
Anti-phospho VE-cadherin (Y731)	Thermo Fisher Scientific
Anti-VE-cadherin	Cell Signaling Technology, Abcam
Anti-α-tubulin	Cell Signaling Technology
HRP-conjugated mouse anti-rabbit	Cell Signaling Technology
Donkey anti-rabbit Alexa Fluor 594 antibody	Abcam
Goat anti-rabbit Alexa Fluor 488 antibody	Abcam
Goat anti-rabbit Alexa Fluor 647-antibody	Abcam
Anti-Aβ (6E10)	Thermo Fisher Scientific
Anti-VE-cadherin Antibody, clone BV6	Sigma-Aldrich
Phalloidin-iFluor 488 Reagent	Abcam
Rabbit anti-VE-cadherin antibody (NBP3-21223)	Novus Biologicals
Anti-APP	Novus Biologicals
RAGE antibody	ABclonal
IgG	Sigma-Aldrich
Phalloidin-iFluor 488	Abcam
Anti-oligomer Aβ (A11)	Thermo Fisher Scientific
Anti-ZO-1	Cell Signaling Technology
Anti-occludin	Cell Signaling Technology

Anti-claudin-5	Cell Signaling Technology

Supplementary Table 3. Details of steered discrete molecular dynamics simulation conditions consisting of the number of independent runs (N_{run}), dimension, time for each run, and accumulative time.

	EC1 dimer	EC1 dimer + $A\beta_m$	EC1 dimer + $A\beta_o$	EC1 dimer + $A\beta_s$
Dimension	1500 × 450 ×1500	1500 × 450 ×1500	1500 × 450 ×1500	1500 × 450 ×1500
N_{run}	70	70	70	70
Time	100 ns	100 ns	100 ns	100 ns
Force windows	7 (0pN, 10pN, 20pN, 30pN, 40pN, 50pN, 60pN)	7 (0pN, 10pN, 20pN, 30pN, 40pN, 50pN, 60pN)	7 (0pN, 10pN, 20pN, 30pN, 40pN, 50pN, 60pN)	7 (0pN, 10pN, 20pN, 30pN, 40pN, 50pN, 60pN)
Accumulative time	49 µs	49 µs	49 µs	49 µs

Supplementary Table 4. Dissociation rate of cadherin dimer under different forces in both presence and absence of Aβ aggregation species in sDMD simulation. The dissociate time is averaged over all independence runs for each case.

Force	Dimer dissociation rate (ns^{-1})			
	EC1 dimer	EC1 dimer + $A\beta_m$	EC1 dimer + $A\beta_o$	EC1 dimer + $A\beta_s$
0pN	0.01065	0.0103	0.0139	0.01134
10pN	0.01162	0.0118	0.0140	0.01271
20pN	0.01622	0.0162	0.0205	0.01814
30pN	0.02317	0.0233	0.0266	0.02447
40pN	0.02938	0.0314	0.0396	0.03754
50pN	0.03817	0.0545	0.0484	0.04032
60pN	0.05739	0.0458	0.0486	0.05295

Supplementary References

1. Jackson, M., Mantsch, H. H. The use and misuse of ftir spectroscopy in the determination of protein structure. *Crit. Rev. Biochem. Mol. Biol.* **30**, 95-120 (1995).
2. Andreasen, M., *et al.* Physical determinants of amyloid assembly in biofilm formation. *mBio* **10**, e02279-02218 (2019).
3. Huma, Z.-e., *et al.* Nanosilver mitigates biofilm formation via fapc amyloidosis inhibition. *Small* **16**, 1906674 (2020).
