# Supplementary material for: Endothelial leakiness elicited by amyloid protein aggregation
Source: Nat Commun. 2024 Jan 19;15:613. doi: 10.1038/s41467-024-44814-1 (PMC10798980; doi:10.1038/s41467-024-44814-1)
Supplement: Supplementary file 3 — Reporting Summary [file 41467_2024_44814_MOESM3_ESM.pdf]

nature portfolio

Corresponding author(s): Pu Chun Ke

Last updated by author(s): Dec 14, 2023

Reporting Summary

Nature Portfolio wishes to improve the reproducibility of the work that we publish. This form provides structure for consistency and transparency in reporting. For further information on Nature Portfolio policies, see our Editorial Policies and the Editorial Policy Checklist.

Statistics

For all statistical analyses, confirm that the following items are present in the figure legend, table legend, main text, or Methods section.

n/a	Confirmed	
☐	☒	The exact sample size (*n*) for each experimental group/condition, given as a discrete number and unit of measurement
☐	☒	A statement on whether measurements were taken from distinct samples or whether the same sample was measured repeatedly
☐	☒	The statistical test(s) used AND whether they are one- or two-sided<br>*Only common tests should be described solely by name; describe more complex techniques in the Methods section.*
☒	☐	A description of all covariates tested
☒	☐	A description of any assumptions or corrections, such as tests of normality and adjustment for multiple comparisons
☐	☒	A full description of the statistical parameters including central tendency (e.g. means) or other basic estimates (e.g. regression coefficient) AND variation (e.g. standard deviation) or associated estimates of uncertainty (e.g. confidence intervals)
☐	☒	For null hypothesis testing, the test statistic (e.g. *F*, *t*, *r*) with confidence intervals, effect sizes, degrees of freedom and *P* value noted<br>*Give P values as exact values whenever suitable.*
☒	☐	For Bayesian analysis, information on the choice of priors and Markov chain Monte Carlo settings
☒	☐	For hierarchical and complex designs, identification of the appropriate level for tests and full reporting of outcomes
☒	☐	Estimates of effect sizes (e.g. Cohen's *d*, Pearson's *r*), indicating how they were calculated

Our web collection on statistics for biologists contains articles on many of the points above.

Software and code

Policy information about availability of computer code

Data collection	Mice tissue fluorescence data collected using EvolutionCapt-v18.02 and Living Image® 4.3.1 software. Protein data bank (PDB) files were collected using the program of Molecules in action, LLC.
Data analysis	ImageJ 1.53c, GraphPad Prism 9.3.1, Python, MATLAB_R2017a, pyMol 2.5.5, Visual Molecular Dynamics (VMD 19.3), Origin 8.5.0 and grace 5.0.5 were used to perform the data analysis. The discrete molecular dynamics (DMD) simulation code and analysis script used to generate the results reported in this manuscript are available at: Molecular in Action, LLC, moleculesinaction.com/home.html and https://doi.org/10.5281/zenodo.10152860.

For manuscripts utilizing custom algorithms or software that are central to the research but not yet described in published literature, software must be made available to editors and reviewers. We strongly encourage code deposition in a community repository (e.g. GitHub). See the Nature Portfolio guidelines for submitting code & software for further information.

Data

Policy information about availability of data

All manuscripts must include a data availability statement. This statement should provide the following information, where applicable:

- Accession codes, unique identifiers, or web links for publicly available datasets
- A description of any restrictions on data availability
- For clinical datasets or third party data, please ensure that the statement adheres to our policy

The source data underlying Figs. 1-6 and Supplementary Figures 1-4, 6, 8-14, 16-20, 25, 26 are provided in a Source Data file. The simulations data for Fig. 7 and

Supplementary Figures 21-24 are deposited to Zenodo link as follows: https://doi.org/10.5281/zenodo.10152860.
The atomic models of EC1 cadherin dimer, Aβm, Aβs are available from the database PDB ID: “3PPE [https://doi.org/10.2210/pdb3PPE/pdb]”, “1IYT [https://doi.org/10.2210/pdb1IYT/pdb]” and “5OQV [https://doi.org/10.2210/pdb5OQV/pdb]”, respectively.

Research involving human participants, their data, or biological material

Policy information about studies with human participants or human data. See also policy information about sex, gender (identity/presentation), and sexual orientation and race, ethnicity and racism.

Reporting on sex and gender	N/A
Reporting on race, ethnicity, or other socially relevant groupings	N/A
Population characteristics	N/A
Recruitment	N/A
Ethics oversight	N/A

Note that full information on the approval of the study protocol must also be provided in the manuscript.

Field-specific reporting

Please select the one below that is the best fit for your research. If you are not sure, read the appropriate sections before making your selection.

[x] Life sciences   [ ] Behavioural & social sciences   [ ] Ecological, evolutionary & environmental sciences

For a reference copy of the document with all sections, see nature.com/documents/nr-reporting-summary-flat.pdf

Life sciences study design

All studies must disclose on these points even when the disclosure is negative.

Sample size	Sample size was determined based on animal availability and accepted sample sizes for these type of experiments. The in vivo studies were performed with at least 3 mice per group while adhering to our approved Southwest University Animal Care and Use Committee standard protocols and minimizing the number of animals to still achieve a statistical validity. For in vitro experiments, previous published results, complexity, and past experience were used to determine the sample size. All our cell experiments described in this paper were performed at least with n = 3. A minimum of 3 independent repeats was required to evaluate statistical significance. Details regarding sample sizes and statistical tests of all experiments are provided in the Methods section and figure captions.
Data exclusions	We did not exclude any data.
Replication	The in vitro cell experiments were repeated at least 3 times, and the results were consistent with each other. For the in vivo work, each group contained at least 3 mice, all data were displayed in the paper and all attempts at replication were successful.
Randomization	Randomization was relevant in our class in the mice experiments. But they were all aged and sex matched before randomization into the various treatment groups. For in vitro studies, same batch of cells were plated and the wells were grouped randomly for each experimental parameter.
Blinding	The sub team analysing the mouse data was independent from the sub team working on the mouse experiments and was blinded from the experimental conditions/groups. In other experiments, samples were analyzed in a blinded manner without subjective estimation.

Reporting for specific materials, systems and methods

We require information from authors about some types of materials, experimental systems and methods used in many studies. Here, indicate whether each material, system or method listed is relevant to your study. If you are not sure if a list item applies to your research, read the appropriate section before selecting a response.

Materials & experimental systems

n/a	Involved in the study	
	x	Antibodies
	x	Eukaryotic cell lines
x		Palaeontology and archaeology
	x	Animals and other organisms
x		Clinical data
x		Dual use research of concern
x		Plants

Methods

n/a	Involved in the study	
x		ChIP-seq
x		Flow cytometry
x		MRI-based neuroimaging

Antibodies

Antibodies used

VE-Cadherin polyclone antibody (Abcam; Product number: ab33168), 1:400 dilution
Donkey anti-rabbit Alexa Fluor 594 antibody (Abcam, Product number: ab150076), 1:500 dilution
Goat anti-rabbit Alexa Fluor 488 antibody (Abcam, Product number: ab150089), 1:500 dilution
Anti-Aβ (6E10) (Thermo Fisher Scientific, Product number: MA5-48043), 1:250 dilution
Anti-oligomer Aβ (A11) (Thermo Fisher Scientific, Product number: AHB0052), 1:250 dilution
RAGE antibody (ABclonal, Product number: A23423), 1:400 dilution
Anti-VE-cadherin antibody, clone BV6 (Sigma-Aldrich, Product number: MABT134), 1:20, 1:100, 1:500 dilution
Anti-VE-cadherin antibody, EC1a (Novus Biologicals; Product number: NBP3-21223), 1:20, 1:100, 1:500 dilution
Phalloidin-iFluor 488 (Abcam, product number: ab176753), 1:500 dilution
Anti-caspase 3 (Cell Signaling Technology; product number 9662), 1:1000 dilution
Anti-caspase 9 (Cell Signaling Technology; product number 9502), 1:1000 dilution
Anti-PARP (Cell Signaling Technology; product number 9542), 1:1000 dilution
Anti-phospho VE-cadherin (Y658) (Thermo Fisher Scientific; product number: 44-1144G), 1:1000 dilution
Anti-phospho VE-cadherin (Y731) (Thermo Fisher Scientific; product number: 44-1145G), 1:1000 dilution
Anti-VE-cadherin (Cell Signaling Technology; product number: 2500), 1:1000 dilution
Anti-α-tubulin (Cell Signaling Technology; product number: 2144), 1:1000 dilution
Anti-rabbit IgG, HRP-linked antibody (Cell Signaling Technology; product number: 7074), 1:1000 dilution
Anti-APP (6E10) (Novus Biologicals; product number: NBP2-62566), 1:500 dilution
Anti-oligomer A11 Polyclonal Antibody (Thermo Fisher Scientific; product number: AHB0052), 1:1000 dilution
Anti-ZO-1 (Cell Signaling Technology; product number: 8193); 1:1000 dilution
Anti-occludin (Cell Signaling Technology; product number: 91131); 1:1000 dilution
Anti-claudin-5 (Cell Signaling Technology; product number: 49564); 1:1000 dilution

Validation

Antibodies were validated by the manufacturer as stated on the website blow: VE-Cadherin polyclone antibody (Abcam; Product number: ab33168) [https://www.abcam.com/products/primary-antibodies/ve-cadherin-antibody-intercellular-junction-marker-ab33168.html]; Donkey anti-rabbit Alexa Fluor 594 antibody (Abcam, Product number: ab150076) [https://www.abcam.com/products/secondary-antibodies/donkey-rabbit-igg-hl-alexa-fluor-594-ab150076.html]; Goat anti-rabbit Alexa Fluor 488 antibody (Abcam, Product number: ab150089) [https://www.abcam.com/products/secondary-antibodies/goat-rabbit-igg-fc-alexa-fluor-488-ab150089.html]; Anti-Aβ (6E10) (Thermo Fisher Scientific, Product number: MA5-48043) [https://www.thermofisher.cn/cn/zh/antibody/product/beta-Amyloid-Chimeric-Antibody-clone-6E10-Recombinant-Monoclonal/MA5-48043]; Anti-oligomer Aβ (A11) (Thermo Fisher Scientific, Product number: AHB0052) [https://www.thermofisher.cn/cn/zh/antibody/product/Oligomer-A11-Antibody-Polyclonal/AHB0052]; RAGE antibody (ABclonal, Product number: A23423) [https://abclonal.com/catalog-antibodies/AGERRabbitmAb/A23423]; Anti-VE-cadherin antibody, clone BV6 (Sigma-Aldrich, Product number: MABT134) [https://www.sigmaaldrich.com/US/en/product/mm/mabt134]; Anti-VE-cadherin antibody, EC1a (Novus Biologicals; Product number: NBP3-21223) [https://www.novusbio.com/products/ve-cadherin-antibody_nbp3-21223]; Phalloidin-iFluor 488 (Abcam, product number: ab176753) [https://www.abcam.com/products/chip-kits/phalloidin-ifluor-488-reagent-ab176753.html]; Anti-caspase 3 (Cell Signaling Technology; product number 9662) [https://www.cellsignal.com/products/primary-antibodies/caspase-3-antibody/9662]; Anti-caspase 9 (Cell Signaling Technology; product number 9502) [https://www.cellsignal.com/products/primary-antibodies/caspase-9-antibody/9502]; Anti-PARP (Cell Signaling Technology; product number 9542) [https://www.cellsignal.com/products/primary-antibodies/parp-antibody/9542]; Anti-phospho VE-cadherin (Y658) (Thermo Fisher Scientific; product number: 44-1144G) [https://www.thermofisher.cn/cn/zh/antibody/product/Phospho-VE-cadherin-Tyr658-Antibody-Polyclonal/44-1144G]; Anti-phospho VE-cadherin (Y731) (Thermo Fisher Scientific; product number: 44-1145G) [https://www.thermofisher.cn/cn/zh/antibody/product/Phospho-VE-cadherin-Tyr731-Antibody-Polyclonal/44-1145G]; Anti-VE-cadherin (Cell Signaling Technology; product number: 2500) [https://www.cellsignal.com/products/primary-antibodies/ve-cadherin-d87f2-xp-rabbit-mab/2500]; Anti-α-tubulin (Cell Signaling Technology; product number: 2144) [https://www.cellsignal.com/products/primary-antibodies/a-tubulin-antibody/2144]; Anti-rabbit IgG, HRP-linked antibody (Cell Signaling Technology; product number: 7074) [https://www.cellsignal.com/products/secondary-antibodies/anti-rabbit-igg-hrp-linked-antibody/7074]; Anti-APP (6E10) (Novus Biologicals; product number: NBP2-62566) [https://www.novusbio.com/products/app-antibody-6e10_nbp2-62566]; Anti-oligomer A11 Polyclonal Antibody (Thermo Fisher Scientific; product number: AHB0052) [https://www.thermofisher.cn/cn/zh/antibody/product/Oligomer-A11-Antibody-Polyclonal/AHB0052]; Anti-ZO-1 (Cell Signaling Technology; product number: 8193) [https://www.cellsignal.com/products/primary-antibodies/zo-1-d7d12-rabbit-mab/8193]; Anti-occludin (Cell Signaling Technology; product number: 91131) [https://www.cellsignal.com/products/primary-antibodies/occludin-e6b4r-rabbit-mab/91131]; Anti-claudin-5 (Cell Signaling Technology; product number: 49564) [https://www.cellsignal.com/products/primary-antibodies/claudin-5-e8f3d-rabbit-mab/49564].

Eukaryotic cell lines

Policy information about cell lines and Sex and Gender in Research

Cell line source(s)	Human dermal microvascular endothelial cells (HMVECs, catalog number: CC-2543) were obtained from Lonza (Switzerland) and hCMEC/D3 (catalog number: SCC066) human brain endothelial cells were derived from Merck Millipore (USA). Sex was not considered in the cell assays.
Authentication	We did not authenticate the cell lines because they were all from commercial sources and they had their own individual companies' quality control policies.
Mycoplasma contamination	We did not test for mycoplasma contamination because the source company had already tested for mycoplasma.
Commonly misidentified lines (See ICLAC register)	None

Animals and other research organisms

Policy information about studies involving animals; ARRIVE guidelines recommended for reporting animal research, and Sex and Gender in Research

Laboratory animals	In total of 148 10-week-old Swiss, seven 2-month-old APP/PS1 mice, six 12-month-old APP/PS1 mice, seven 2-month-old C57BL/6J mice, and six 12-month-old C57BL/6J mice were used. The mice were supplied with free access to food and water and were kept at 22 ± 2 ℃ with 50 ± 10% humidity environment, and light/dark cycle of 12 h.
Wild animals	No wild animals were used in the study.
Reporting on sex	Both sexes were used in this study. We have measured the vascular leakiness in vivo induced by Aβ species. An equal number of male and female mice/per group were used for this assay, data for 3 male mice per group were shown in Figure 6C and data for 3 female mice per group were shown in Supplementary Figure 15. No difference was observed in the results of the two experiments. For the other in vivo assays, including data of Figures 6A&F and Supplementary Figures 14, 17A, 19A&B,20A&B, the sex of the mice was randomly selected.
Field-collected samples	No field collected samples were used in the study.
Ethics oversight	Southwest University Animal Care and Use Committee.

Note that full information on the approval of the study protocol must also be provided in the manuscript.
